# AI-Driven segmentation and morphogeometric profiling of epicardial adipose tissue in type 2 diabetes

**DOI:** 10.1186/s12933-025-02829-y

**Published:** 2025-07-18

**Authors:** Fan Feng, Abdallah I. Hasaballa, Ting Long, Xinyi Sun, Justin Fernandez, Carl-Johan Carlhäll, Jichao Zhao

**Affiliations:** 1 Auckland Bioengineering Institute, The University of Auckland, 70 Symonds Street, Auckland, 1010 New Zealand; 2 Department of Computer Science, University of Oxford, Oxford, UK; 3 Faculty of Medical and Health Sciences, School of Medicine, The University of Auckland, Auckland, New Zealand; 4 Center for Medical Image Science and Visualization (CMIV), Linköping University, Linköping, Sweden; 5 Department of Clinical Physiology in Linköping, Department of Health, Medicine and Caring Sciences, Linköping University, Linköping, Sweden; 6 Department of Engineering Science and Biomedical Engineering, The University of Auckland, Auckland, New Zealand

**Keywords:** Epicardial adipose tissue, Type 2 diabetes, MRI, Multi-modal deep learning, Statistical shape analysis

## Abstract

**Background:**

Epicardial adipose tissue (EAT) is associated with cardiometabolic risk in type 2 diabetes (T2D), but its spatial distribution and structural alterations remain understudied. We aim to develop a shape-aware, AI-based method for automated segmentation and morphogeometric analysis of EAT in T2D.

**Methods:**

A total of 90 participants (45 with T2D and 45 age-, sex-matched controls) underwent cardiac 3D Dixon MRI, enrolled between 2014 and 2018 as part of the sub-study of the Swedish SCAPIS cohort. We developed EAT-Seg, a multi-modal deep learning model incorporating signed distance maps (SDMs) for shape-aware segmentation. Segmentation performance was evaluated using the Dice similarity coefficient (DSC), the 95% Hausdorff distance (HD95), and the average symmetric surface distance (ASSD). Statistical shape analysis combined with partial least squares discriminant analysis (PLS-DA) was applied to point cloud representations of EAT to capture latent spatial variations between groups. Morphogeometric features, including volume, 3D local thickness map, elongation and fragmentation index, were extracted and correlated with PLS-DA latent variables using Pearson correlation. Features with high-correlation were identified as key differentiators and evaluated using a Random Forest classifier.

**Results:**

EAT-Seg achieved a DSC of 0.881, a HD95 of 3.213 mm, and an ASSD of 0.602 mm. Statistical shape analysis revealed spatial distribution differences in EAT between T2D and control groups. Morphogeometric feature analysis identified volume and thickness gradient-related features as key discriminators (*r* > 0.8, *P* < 0.05). Random Forest classification achieved an AUC of 0.703.

**Conclusions:**

This AI-based framework enables accurate segmentation for structurally complex EAT and reveals key morphogeometric differences associated with T2D, supporting its potential as a biomarker for cardiometabolic risk assessment.

**Graphical abstract:**

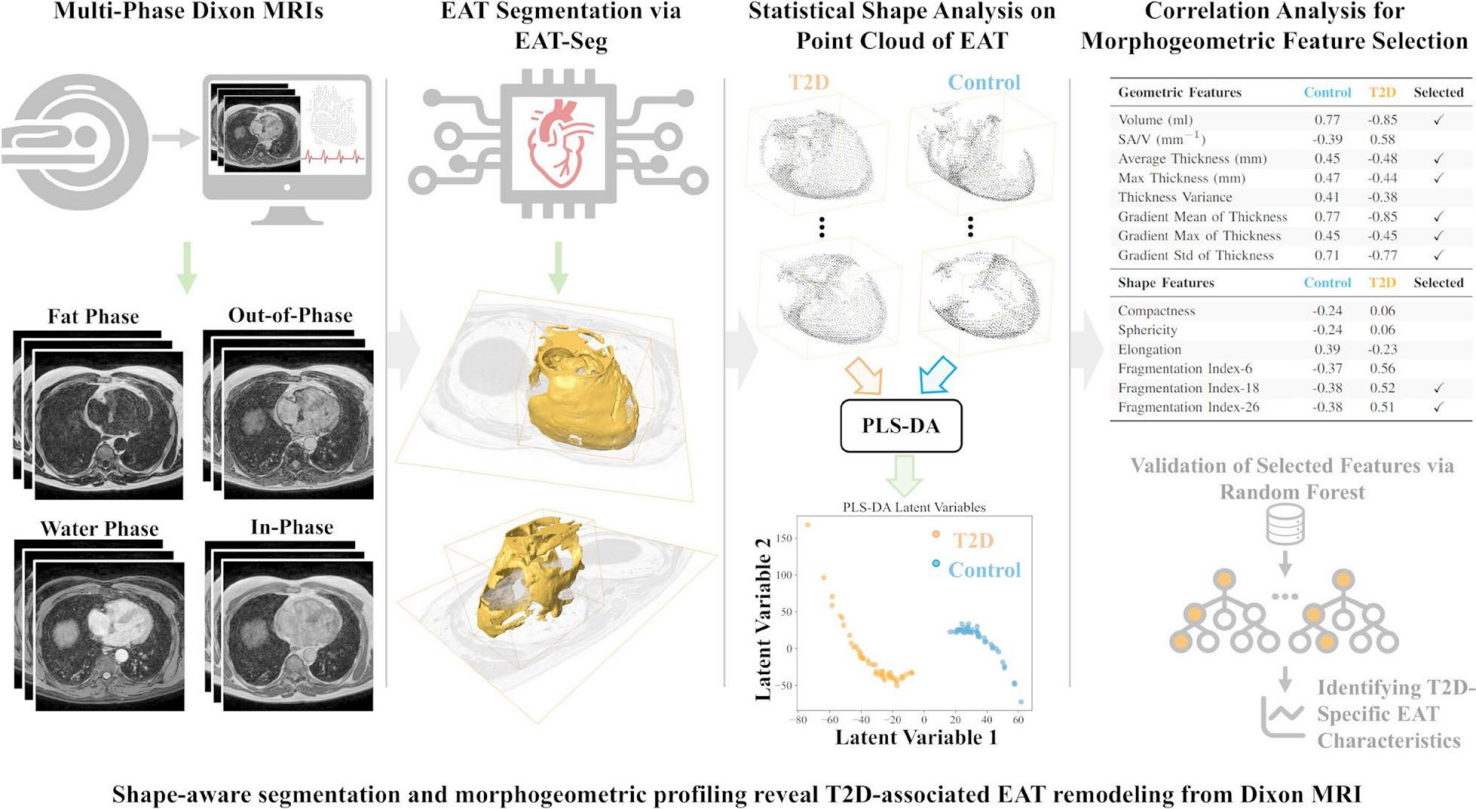

**Supplementary Information:**

The online version contains supplementary material available at 10.1186/s12933-025-02829-y.

## Introduction

Patients with type 2 diabetes (T2D) are at higher risk of developing cardiovascular disease (CVD) and heart failure compared to non-diabetic individuals [1, 2]. Understanding of the underlying pathophysiology and identifying novel imaging biomarkers are crucial for early detection and risk stratification [3]. Epicardial adipose tissue (EAT), a metabolically active fat depot located between the myocardium and pericardium, has gained recognition due to its close anatomical and functional relationship with the heart [4, 5]. Unlike other visceral fat depots, EAT is uniquely situated adjacent to the myocardium without fascial separation and receives blood supply from the coronary circulation, enabling direct paracrine and vasocrine interactions [6]. In T2D, EAT undergoes pathological remodeling and contributes to cardiovascular risk through multiple mechanisms, including increased inflammation, autonomic dysregulation, and mechanical compression from enlarged fibrotic fat pads [7–9]. It also secretes proinflammatory and profibrotic cytokines that can impair myocardial function, promote fibrosis, and accelerate atherosclerosis. Consequently, pathological changes in EAT, such as increased volume and altered geometry, have been associated with exacerbated cardiovascular risks in T2D patients [10].

Magnetic resonance imaging (MRI), particularly Dixon water-fat separation imaging, offers excellent soft tissue contrast and enables accurate characterization of fat distribution. Dixon MRI generates multiple phase images, including in-phase (IP), out-of-phase (OP), fat phase (FP), and water phase (WP) images [11, 12], enhancing tissue differentiation and making it well-suited for the assessment of EAT. Despite these advantages, segmentation of EAT on MRI remains labor-intensive, relying heavily on manual delineation due to the structurally complex and irregular anatomy of EAT. Previous studies investigating EAT have primarily focused on computed tomography (CT) imaging [13–16], with only limited MRI-based investigations [17, 18].

Recent advances in medical image analysis have demonstrated the effectiveness of deep learning-based segmentation models, such as U-Net, for EAT segmentation. These approaches typically employ a two-stage convolutional neural network (CNN) architecture that first localizes the region containing thoracic adipose tissue and then segments EAT [14, 19]. However, they often lack shape-awareness, limiting segmentation accuracy in anatomically complex regions. While several MRI-based studies have also proposed deep learning methods for EAT segmentation, they are hindered by low through-plane resolution (i.e., greater slice thickness) of MRI datasets, which makes it difficult to obtain detailed and accurate 3D EAT representations, posing challenges for further shape and geometric analysis [17, 18, 20]. Appendix Table S2 summarizes published studies on deep learning-based EAT segmentation and analysis.

In the quantitative assessment of EAT [21, 22], most studies have relied on volumetric measurements to estimate global fat accumulation [23, 24], but these fail to characterize critical spatial and morphogeometric features including shape, regional distribution, and tissue heterogeneity. Recognizing this, recent studies have explored EAT thickness as an alternative metric [25–27]. However, these assessments are often restricted to simplified measurements such as maximum or localized thickness [28], which do not adequately reflect the full spatial complexity or morphological variability of EAT. Advanced approaches such as statistical shape analysis offer the potential to comprehensively characterize geometric variations and identify morphological patterns across populations [29]. Despite their successful application to other anatomical structures, these methods have not yet been applied to EAT, leaving a significant opportunity for deeper phenotypic characterization.

**Fig. 1:**
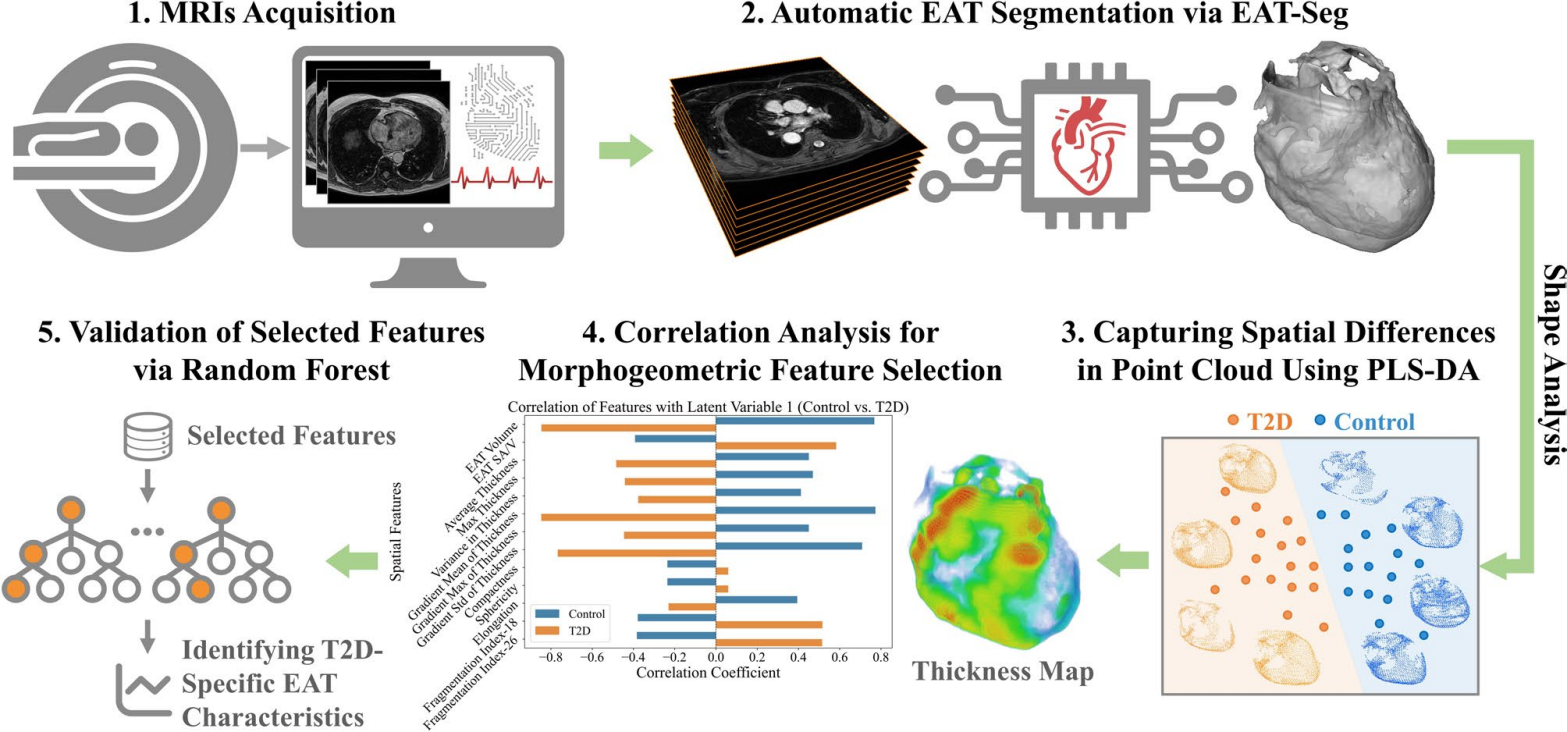
Overview of the epicardial adipose tissue (EAT) segmentation and analysis pipeline for investigating morphogeometric differences between type 2 diabetes (T2D) and control groups. The workflow begins with Dixon MRI acquisition, followed by automatic EAT segmentation using a deep learning-based method (EAT-Seg). Statistical shape analysis is performed on point cloud representations of EAT across all samples. Partial least squares-discriminant analysis (PLS-DA) is applied to capture spatial differences between groups through latent variables. Morphogeometric features, including geometric and shape-based metrics, are extracted and analyzed for their correlation with latent variables. Features showing high correlations are identified as key contributors to group separation. Random Forest model is used to evaluate these features for identifying T2D-specific EAT characteristics

To bridge these gaps, the purpose of this study was to develop an AI-driven framework for automated EAT segmentation and morphogeometric analysis of EAT from Dixon MRI. This approach enables a detailed investigation of EAT geometry and shape differences between T2D patients and controls (Fig. 1). Our key contributions include:

- *EAT-Seg*: We introduce a multi-modal state-space model (SSM)-based deep learning model for shape-aware segmentation of EAT, leveraging multiple phase images from Dixon MRI to enhance anatomical accuracy.
- *Statistical Shape Analysis*: We apply statistical shape analysis to point cloud representations of EAT, enabling population-level differentiation between T2D and control groups based on latent shape features while capturing spatial distribution variability.
- *Morphogeometric Feature Extraction*: We extract morphogeometric features, including EAT volume, local thickness distribution, elongation and fragmentation indices. These features are further evaluated using a Random Forest classifier to identify T2D-specific EAT characteristics.

This work provides new insights into the morphogeometric remodeling of EAT in T2D and supports the exploration of EAT characteristics as potential imaging biomarkers for cardiometabolic disease.

## Methods

### Study population

**Fig. 2:**
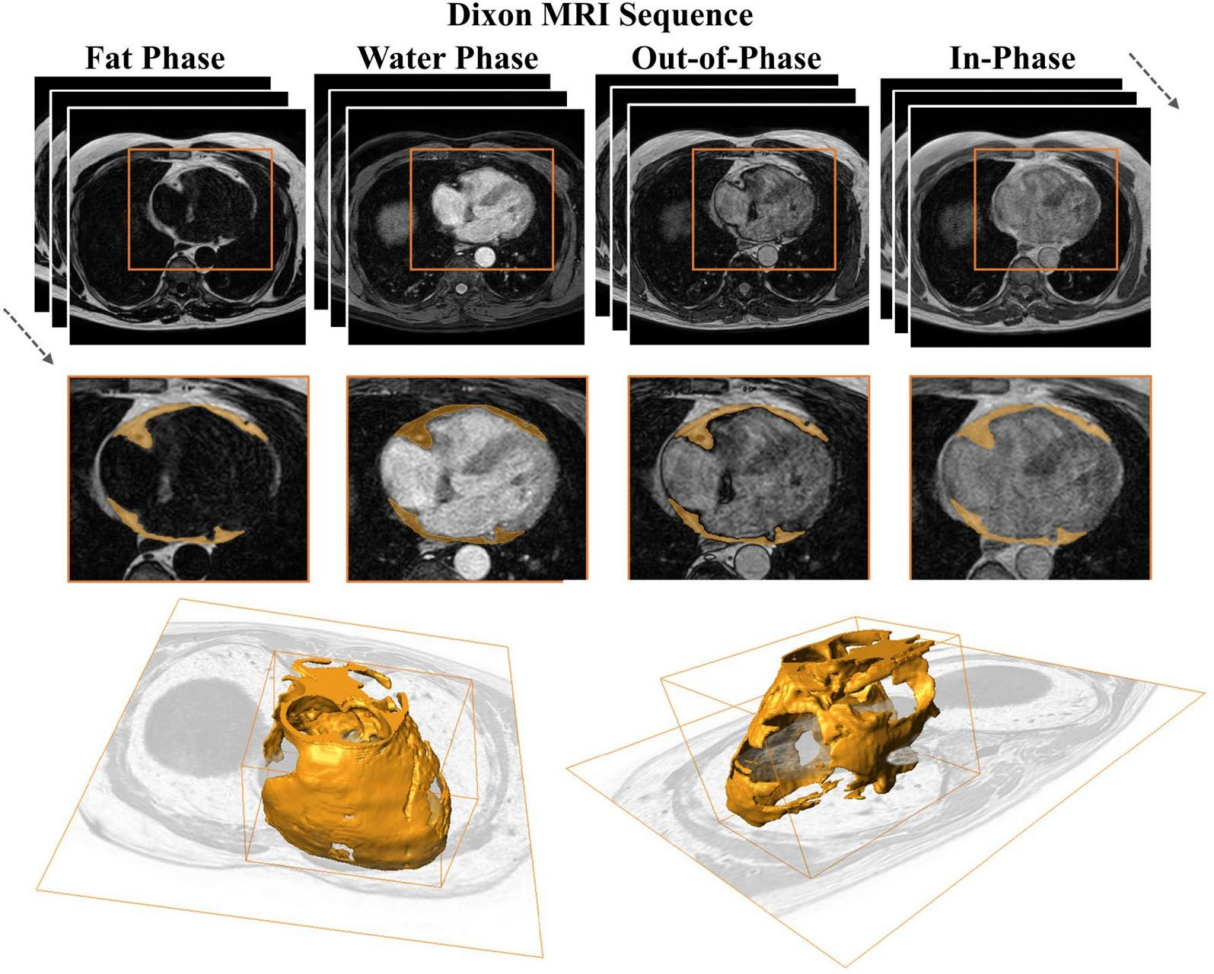
The Dixon MRI sequence consists of four phases: fat phase (FP), water phase (WP), out-of-phase (OP) and in-phase (IP) images. Each phase contains the same number of slices. Epicardial adipose tissue (EAT) is identified as interior pixels highlighted in orange. The different phases show varying contrasts that contain both structural and contextual information. 3D reconstruction of EAT segmentation from Dixon MRI is shown in the bottom row

Experiments were conducted on a Dixon MRI dataset from a sub-study (HEALTH) of the Swedish CArdioPulmonarybiolmage Study (SCAPIS) [30, 31], a large, prospective, population-based cohort aimed at improving risk prediction of cardiopulmonary and metabolic diseases and optimizing the ability to study disease mechanisms. SCAPIS recruited individuals aged 50–64 years, randomly selected from the Swedish population register across six university hospitals. In our study, we included 90 subjects: 45 with T2D and 45 controls matched according to age (50–64) and sex (35.6% female in both groups). Patient characteristics are detailed in Appendix Table S1. Participants followed the general inclusion and exclusion criteria of SCAPIS; the only exclusion criterion was the inability to understand spoken and written Swedish, as this precluded provision of informed consent. No additional clinical exclusion criteria were applied, ensuring broad population representativeness. All participants provided written informed consent. The study complied with the Declaration of Helsinki and was approved by the Linköping Ethical Review Board (Dnr 2023-02089-02). Images were reconstructed at a spatial resolution of 0.67 mm × 0.67 mm × 1.5 mm. Each three-dimensional (3D) Dixon MRI dataset comprises 50 to 82 slices along the Z-axis, with an in-plane dimension of 448 × 448 pixels (Fig. 2). Annotations were performed by two experts, each with over three years of experience in cardiac MRI interpretation and atrial anatomy. To ensure label accuracy, a third expert reviewed the annotations, and any disagreements were resolved through consensus discussions among the three experts (Appendix S1).

### Architecture of EAT-Seg

**Fig. 3:**
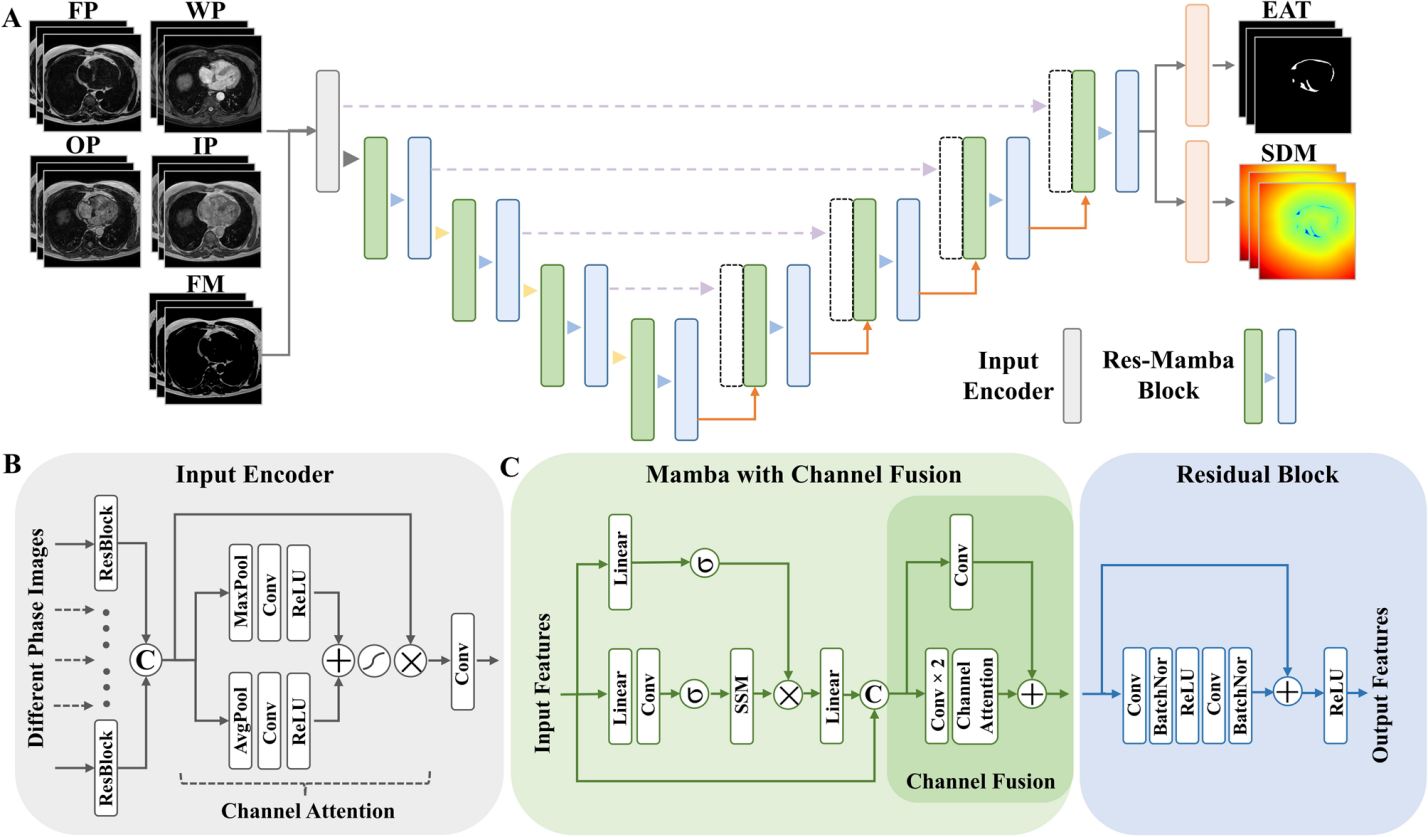
Overview of the EAT-Seg architecture and its components.** A** the model follows a U-shaped architecture with Res-Mamba blocks in the encoder and decoder paths, processing multi-phase Dixon MRI inputs to generate epicardial adipose tissue (EAT) segmentation masks and signed distance maps (SDMs).** B** the input encoder integrates multi-phase Dixon MRI data using an input-level fusion strategy and a channel attention mechanism to emphasize key features.** C** the Res-Mamba block enhances feature extraction by combining long-range dependencies (SSM-based Mamba block), multi-scale integration (channel fusion layer), and fine-grained feature preservation (Residual Block), improving segmentation in anatomically complex EAT regions

To achieve automated and accurate segmentation of EAT, we developed EAT-Seg, a deep learning model based on a U-shaped SSM architecture designed to effectively capture multi-modal and multi-scale image features. EAT-Seg leverages the unique properties of Dixon MRI, which provides four different phase images. The model incorporates a multi-head design with outputs for both binary segmentation and signed distance map (SDM) prediction, enhancing segmentation performance by learning both shape and boundary details (Fig. 3A).

#### Input encoder

The input encoder adopts an input-level fusion strategy to integrate information from multiple phase images of Dixon MRI (Fig. 3B), including IP, OP, FP, WP, and a denoised fat map (FM) derived to enhance fat signal (Appendix S2). This strategy ensures simplicity, computational efficiency, and spatial consistency across phases. Each input channel is processed independently through a Residual Block (ResBlock) to extract robust feature representations. The output of the ResBlock for each channel can be expressed as.

1 $$\mathbf{I}'_m = \mathit{ResBlock}(\mathbf{I}_m), \quad m \in \{\text{IP}, \text{OP}, \text{FP}, \text{WP}, \text{FM}\}$$

where ${{\bf I}}_{m}^{\prime} \in \mathbb{R}^{{~H~ \times ~W~ \times ~D}}$ represents the encoded feature map for each modality. The encoded feature maps from all channels are concatenated along the channel dimension to form a unified multi-modal representation:

2 $$\mathbf{I} = \left[ \mathbf{I}'_{\text{IP}},\ \mathbf{I}'_{\text{OP}},\ \mathbf{I}'_{\text{FP}},\ \mathbf{I}'_{\text{WP}},\ \mathbf{I}'_{\text{FM}} \right]$$

The concatenated tensor **I** is further refined using a channel attention mechanism, which weights the importance of different phases and enhances phase-specific contributions. This ensures that the most relevant information is emphasized while minimizing redundancy, enabling the network to effectively integrate features from different inputs.

#### Res-Mamba block

The Res-Mamba block enhances feature extraction by combining long-range and local contextual information (Fig. 3C). It consists of two main components.

#### Mamba with channel fusion

Mamba, derived from SSMs, captures long-range spatial dependencies through an input-dependent selective mechanism optimized for efficiency [32]. It has been increasingly applied in medical image analysis for its ability to model global contextual information [33]. In EAT-Seg, Mamba is followed by a CNN-based channel fusion block with an attention mechanism to enhance integration of channel-wise features. This design allows the network to leverage both spatial and channel-wise information.

#### Residual block

The ResBlock refines the features extracted by the channel fusion layer, focusing on local contextual information. This block uses convolutional layers with batch normalization and ReLU to extract fine-grained spatial features.

#### Shape-Aware Multi-Head outputs

The model employs a multi-head design to simultaneously predict binary segmentation masks and SDMs. The binary segmentation output provides pixel-wise classification of EAT, while the SDM output improves shape-awareness by predicting the signed distance of each pixel to the nearest boundary. The SDM computation is defined as follows.

3 $$\text{SDM}(x, y) = \begin{cases} d_{\text{neg}}(x, y) - d_{\text{pos}}(x, y),\quad\text{if } (x, y) \notin \text{boundary} \\ 0,\quad\quad\quad\quad\quad\quad\quad\quad\quad\quad \text{if } (x, y) \in \text{boundary} \end{cases}$$

Here, $\:{d}_{pos}\left(x,\:y\right)$ represents the distance from the pixel $\:\left(x,\:y\right)$ to the nearest foreground boundary, and $\:{d}_{neg}\left(x,\:y\right)$ is the distance to the nearest background boundary. Boundary pixels are assigned to zero for normalization. The SDM is then normalized to a range of [− 1,1], ensuring that both positive and negative distances are effectively captured.

### Loss function

The loss function used in the proposed model combines Dice loss, cross-entropy loss, and a regression term for SDM prediction. The overall loss is expressed as:

4 $$\mathcal{L}_{\text{total}} = (1 - \alpha)\mathcal{L}_{\text{Dice+CE}} + \alpha \mathcal{L}_{\text{dist}}$$

where $\:{\mathcal{L}}_{\text{D}\text{i}\text{c}\text{e}+\text{C}\text{E}}$ represents the unweighted sum of Dice loss and cross-entropy loss. The parameter $\:{\upalpha\:}\in\:[0,\:1]$ controls the trade-off between the segmentation and regression components, which was initially set to 0.4. Both L1 and L2 norms are tested for the SDM regression term $\:{\mathcal{L}}_{\text{d}\text{i}\text{s}\text{t}}$ (Appendix S3).

### Statistical shape analysis via PLS-DA from point clouds

Statistical shape analysis aims to capture and differentiate spatial distribution differences in EAT between groups. It was performed on point clouds extracted from EAT volumes. To ensure spatial consistency across samples, preprocessing steps including alignment, non-rigid registration, and feature fitting were applied (Appendix S4). PLS-DA was applied to processed point cloud data to analyze group-specific spatial distribution variations in EAT (Appendix Figure S1). PLS-DA is a multivariate statistical technique that projects high-dimensional feature data onto a lower-dimensional latent space, optimizing group separation.

In this process, each sample’s point cloud was vectorized to form a row in the input matrix (**X**), and the corresponding group label (T2D or control) formed the output vector (**Y**). A two-component PLS-DA model was trained to extract latent variables that captured the primary modes between groups. To further quantify the contribution of specific features to group separation, Variable importance in projection (VIP) scores were calculated for each feature in **X**. VIP scores reflect the relative importance of individual features in explaining the variation captured by the latent variables. Features with VIP scores greater than one were considered significant contributors. High-contribution features were subsequently spatially mapped onto the EAT to visualize regions exhibiting structural variations associated with group differences.

### Morphogeometric feature extraction, selection and modeling

To characterize the geometric and morphological properties of EAT, we extracted a set of biologically and anatomically relevant morphogeometric features aimed at capturing both global EAT distribution and regional heterogeneity. Geometric features included total volume, surface area-to-volume (SA/V) ratio, and voxel-wise thickness metrics derived from local thickness maps [34], such as average thickness, maximum thickness, and thickness variance. To further assess the heterogeneity in EAT distribution, we calculated thickness gradient metrics, including the mean, maximum, and standard deviation of the gradient magnitude of the local thickness map. These descriptors quantify regional variation and non-uniformity in EAT thickness. Shape features characterized the overall morphology and structural complexity of EAT, including compactness, sphericity, and elongation. In addition, to assess the degree of spatial disconnection observed in EAT volumes, we introduced fragmentation indices (-6, -18, -26), defined as the number of disconnected EAT components (connected regions) normalized by total volume (Appendix S5).

To explore the association between PLS-DA-derived latent variables and morphogeometric features, Pearson correlation analysis was performed. Features with high correlation were selected as key indicators of group-specific morphological differences. These selected features were used to interpret abstract latent variables in terms of tangible EAT geometry and shape properties. To further assess whether the selected high-contribution features reliably distinguish EAT characteristics between groups, they were subsequently utilized as inputs to train a Random Forest classifier. The dataset was randomly divided into training (70%) and testing (30%) subsets, with the testing set including 14 control and 13 T2D samples, while maintaining balanced class distributions. Five-fold cross-validation combined with grid search was used to optimize hyperparameters. The final model was trained and evaluated based on receiver operating characteristic (ROC) curve analysis.

### Statistical analyses

All model development and statistical analyses were performed using Python (version 3.10). The EAT-Seg was implemented with Pytorch (version 2.3.1, CUDA 11.8), Mamba-SSM (version 2.0.3), MONAI (version 1.3.1) (implementation details described in Appendix S6). The dataset was divided into training (60%, 54 participants), validation (9%, 8 participants), and testing (31%, 28 participants) subsets. The testing set consisted of matched pairs of T2D and control participants. Segmentation performance was evaluated using Dice similarity coefficient (DSC), 95% Hausdorff distance (HD95), and average symmetric surface distance (ASSD), providing quantitative assessment of segmentation accuracy and boundary agreement. Statistical analysis was conducted using SciPy (version 1.10.1) and Scikit-learn (version 1.3.2). The normality of continuous variables was assessed using the Shapiro-Wilk test. Differences between matched T2D and control groups were analyzed using paired t-tests for normally distributed variables and Wilcoxon signed-rank tests for non-normally distributed variables. A two-sided *P* < 0.05 was considered statistically significant.

## Results

### Dataset characteristics

The study included 90 participants, consisting of 45 individuals with T2D and 45 age-, sex-matched controls. The median age was 60 years (range, 50–64 years), and 35.6% (32/90) of the participants were female (Appendix Table S1).

### Segmentation performance

**Fig. 4:**
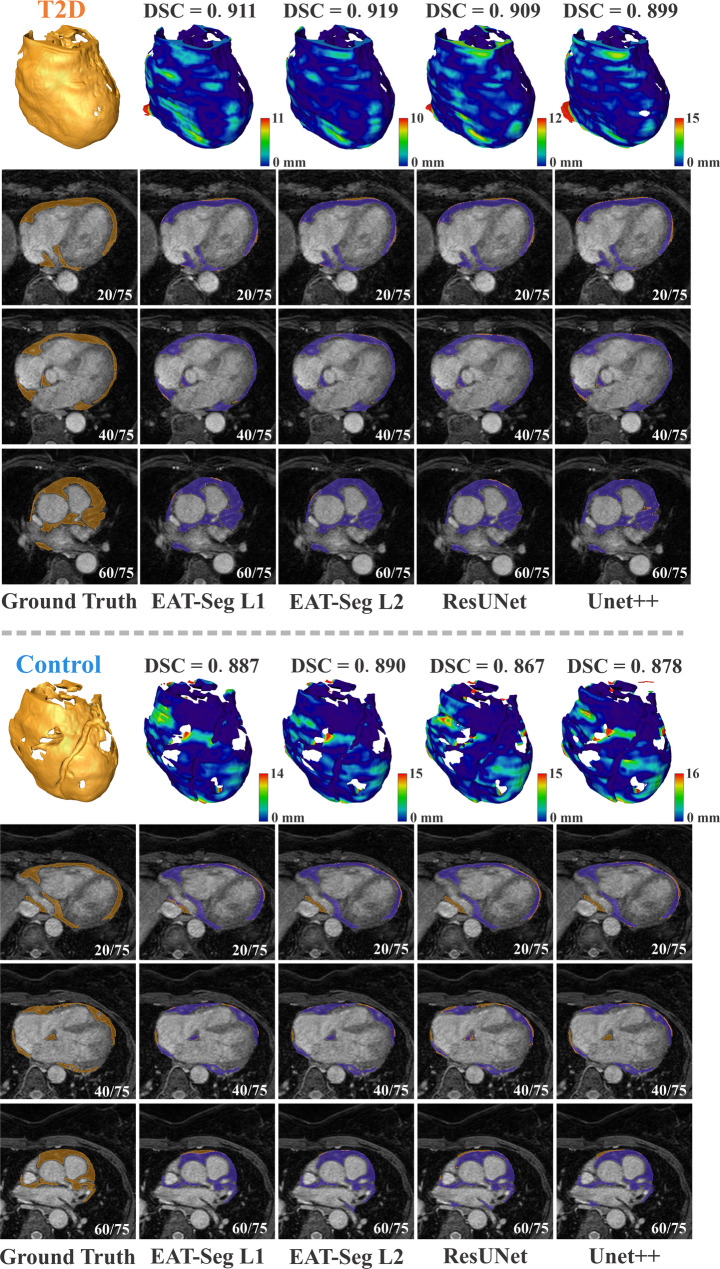
Comparison of EAT-Seg with representative methods. Segmentation results for one T2D and one control subject are visualized in both 3D and 2D slices. The top row presents 3D reconstructions of the ground truth (orange) and surface distance error maps for different methods. The subsequent rows show axial slices at cranial, middle, and caudal regions of the heart, with segmentation overlays. The ground truth is shown in orange, while the predicted results are overlaid in violet. DSC, Dice similarity coefficient; T2D, type 2 diabetes

The segmentation performance of EAT-Seg was compared against ResUNet [35] and UNet++ [36] across all phase combinations. As presented in Table 1, EAT-Seg using both L1 and L2 loss functions (with loss weight parameter α = 0.4) outperformed baseline models in terms of DSC, HD95 and ASSD. Furthermore, using all four Dixon MRI phases, EAT-Seg (L2) achieved the best performance (DSC 0.881, HD95 3.213 mm, ASSD 0.602 mm) compared to the commonly used WP and FP combination (DSC 0.874, HD95 3.512 mm, ASSD 0.678 mm). Comparisons between the L1 and L2 configurations showed no statistically significant differences across all metrics. For the DSC metric, a paired t-test indicated no significant difference (*P* = 0.530), while for the HD95 and ASSD metrics, Wilcoxon signed-rank tests indicated no significant differences (*P* = 0.181 and *P* = 0.295, respectively). Representative segmentation results, including three image slices, 3D reconstructions, and 3D error maps for each patient, are shown in Fig. 4.

**Table 1:** Performance comparison of EAT-Seg (L1/L2), resunet and UNet + + across different phase combinations

Phase				EAT-Seg L1			EAT-Seg L2				ResUNet				UNet++			
**FP**	**WP**	**OP**	**IP**	DSC	HD95	ASSD	DSC	HD95	ASSD	DSC		HD95	ASSD	DSC		HD95	ASSD	
●	●	●	●	**0.880**	**3.468**	**0.586**	**0.881**	**3.213**	**0.602**	0.868		3.461	0.679	0.859		4.515	0.919	
○	●	●	●	0.877	3.562	0.631	0.876	3.634	0.634	0.864		3.420	0.621	0.866		4.067	0.795	
●	●	○	●	0.872	3.462	0.625	0.870	4.055	0.746	0.850		3.684	0.671	0.846		4.446	0.819	
●	○	●	●	0.878	3.342	0.602	0.877	3.533	0.641	0.866		3.250	0.640	0.856		4.261	0.772	
●	●	●	○	0.877	3.464	0.648	0.879	3.448	0.621	0.868		3.366	0.606	0.862		4.075	0.710	
●	●	○	○	0.874	3.512	0.678	0.874	3.332	0.561	0.850		3.514	0.673	0.843		4.314	0.739	
●	○	○	●	0.872	4.152	0.751	0.869	3.568	0.713	0.843		3.320	0.710	0.838		4.343	0.804	
○	○	●	●	0.875	3.961	0.708	0.874	3.541	0.646	0.854		3.979	0.707	0.851		4.457	0.771	
●	○	●	○	0.878	3.513	0.628	0.876	3.494	0.604	0.868		3.074	0.617	0.859		3.977	0.729	
○	●	●	○	0.878	3.433	0.615	0.877	3.320	0.617	0.861		3.476	0.652	0.858		3.711	0.744	
○	●	○	●	0.869	3.646	0.642	0.871	3.621	0.693	0.836		3.873	0.680	0.830		4.594	0.757	
●	○	○	●	0.870	3.125	0.545	0.869	3.346	0.613	0.826		3.927	0.809	0.820		4.483	0.809	
○	○	●	○	0.875	4.080	0.688	0.874	3.558	0.659	0.851		3.845	0.677	0.848		4.420	0.714	
○	●	○	○	0.869	3.846	0.622	0.867	3.507	0.625	0.744		6.307	1.181	0.737		6.469	1.327	
○	○	○	●	0.861	3.613	0.625	0.862	3.935	0.703	0.708		6.233	1.188	0.699		6.395	1.412	

Missing and available modalities are denoted by ○ and ●, respectively. FP, fat phase; WP, water phase; OP, out-of-phase; IP, in-phase; DSC, Dice similarity coefficient; HD95, 95% Hausdorff distance, mm; ASSD, average symmetric surface distance, mm

### Parameter setting of loss functions

The effect of the weighting parameter α on segmentation performance was evaluated by varying α from 0.0 to 0.9. As summarized in Table 2, both L1- and L2-based loss functions achieved the highest DSC values around α = 0.3–0.4, with the best DSC (0.882) obtained using L2. The lowest HD95 and ASSD values were also observed within this range. When α exceeded 0.5, segmentation performance progressively deteriorated across all metrics. Parameter α value between 0.3 and 0.4 provides an optimal balance between shape constraint and segmentation accuracy.

**Table 2:** Effect of α on segmentation performance for L1 and L2

		α = 0.0	α = 0.1	α = 0.2	α = 0.3	α = 0.4	α = 0.5	α = 0.6	α = 0.7	α = 0.8	α = 0.9
**L1**	DSC	0.875	0.876	0.878	0.880	0.880	0.879	0.879	0.878	0.874	0.874
	HD95	3.881	3.694	3.733	**3.208**	3.468	3.534	3.410	3.454	3.618	3.791
	ASSD	0.619	0.662	0.680	**0.580**	0.586	0.656	0.616	0.614	0.638	0.654
**L2**	DSC	0.874	0.879	0.878	**0.882**	0.881	0.877	0.879	0.878	0.878	0.874
	HD95	3.682	3.533	3.611	3.348	3.213	3.484	3.474	3.526	3.622	3.774
	ASSD	0.640	0.657	0.676	0.606	0.602	0.650	0.604	0.625	0.640	0.647

DSC, Dice similarity coefficient; HD95, 95% Hausdorff distance, mm; ASSD, average symmetric surface distance, mm

### Shape analysis via PLS-DA

**Fig. 5:**
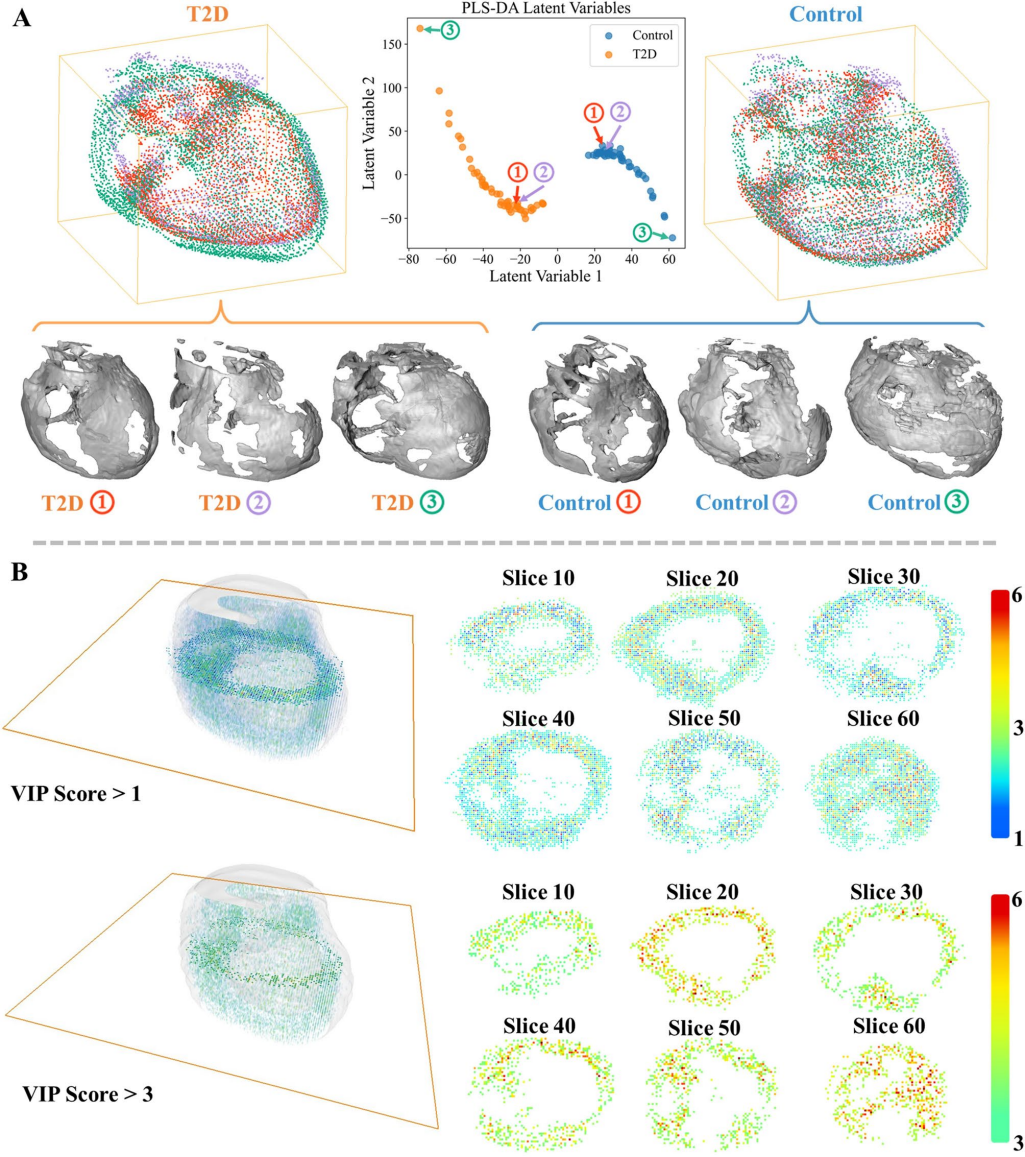
Statistical shape analysis of epicardial adipose tissue (EAT) in type 2 diabetes (T2D) and control groups.** A** PLS-DA separates T2D and control groups based on EAT point cloud data, with Latent Variable 1 distinguishing between groups and Latent Variable 2 capturing intra-group variability. Representative samples from each group are visualized as 3D point clouds (top) and corresponding EAT reconstructions (bottom), highlighting structural differences in EAT morphology.** B** spatial distribution of high-contribution regions identified by variable importance in projection (VIP) scores. Regions with VIP scores exceeding 1 (top) and 3 (bottom) highlight key spatial differences between T2D and control groups

The PLS-DA analysis revealed that latent variable 1 (LV1) effectively distinguished between T2D and control groups based on EAT point cloud data (Fig. 5A, central panel). Samples from T2D participants clustered separately from controls, indicating group-specific spatial distribution differences. Latent Variable 2 (LV2) captures additional with-group variability. Three representative samples per group were selected for detailed visualization, demonstrating that samples close together in latent space had similar EAT distributions, whereas distant samples exhibited distinct morphologies (Fig. 5A, left and right panels). VIP scores were calculated for each spatial point to identify regions contributing most to group separation. Points with VIP scores greater than 1 and greater than 3 are illustrated in Fig. 5B. High-VIP-score regions were distributed throughout the EAT, with greater concentrations in areas of increased thickness.

### Morphogeometric feature selection and modeling

**Fig. 6:**
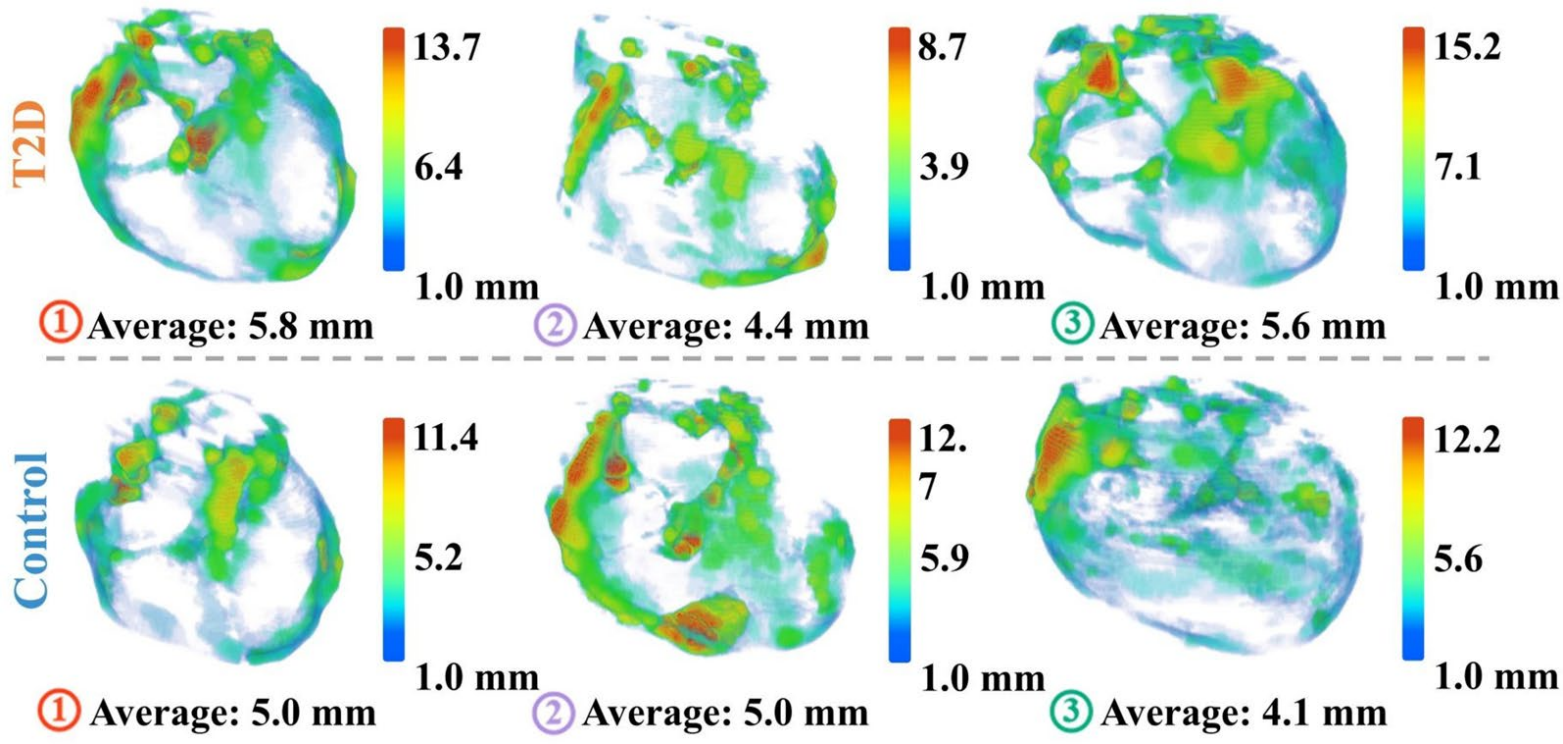
Local thickness maps of epicardial adipose tissue (EAT) for representative samples from type 2 diabetes (T2D) and control groups. These six samples correspond to those shown in Fig. 5A. The color-coded thickness maps illustrate regional variations in EAT thickness. T2D samples exhibit relatively higher average thickness and greater spatial heterogeneity compared to controls

Morphogeometric features provided geometric and shape-based characteristics of EAT in T2D and control groups. As summarized in Table 3, all morphogeometric features exhibited statistically significant differences between the two groups except for Fragmentation Index-6 (*P* = 0.1022). EAT volume was significantly larger in T2D participants compared to controls (*P* = 0.0045). SA/V ratio was significantly lower in T2D participants (*P* = 0.0046). Thickness-related metrics were computed from local thickness map of each EAT volume, as visualized in Fig. 6. Average thickness (*P* = 0.0022), maximum thickness (*P* = 0.0038), and thickness variance (*P* = 0.0078) were all significantly higher in T2D participants. Thickness gradient metrics, including mean, maximum, and standard deviation of thickness gradients, were also significantly higher in the T2D group compared to controls. Elongation was significantly greater in the T2D group (*P* = 0.008). Compactness and sphericity were also higher in T2D participants (*P* = 0.0401 and *P* = 0.0401, respectively). Fragmentation Index-18 (*P* = 0.0013) and Fragmentation Index-26 (*P* = 0.0014) were significantly lower in T2D participants.

**Table 3:** Comparison of geometric and shape features of EAT between T2D and control groups

Geometric features	Control (Mean ± Std)	T2D (Mean ± Std)	*P*-Value
Volume (ml)	101.083 ± 40.2914	127.963 ± 46.2175	**0.0045**
SA/V (mm^− 1^)	0.506 ± 0.0987	0.450 ± 0.1036	**0.0046**
Average thickness (mm)	5.214 ± 0.8567	5.869 ± 1.0907	**0.0022**
Max thickness (mm)	12.173 ± 2.2673	13.836 ± 2.9586	**0.0038**
Thickness variance	6.275 ± 2.8766	8.602 ± 4.6566	**0.0078**
Gradient mean of thickness	0.025 ± 0.0097	0.032 ± 0.0113	**0.0041**
Gradient max of thickness	10.555 ± 1.9315	11.896 ± 2.5930	**0.0069**
Gradient std of thickness	0.271 ± 0.0683	0.319 ± 0.0808	**0.0034**

Shape features	Control (Mean ± Std)	T2D (Mean ± Std)	*P*-Value
Compactness	0.030 ± 0.0031	0.031 ± 0.0032	**0.0401**
Sphericity	0.145 ± 0.0148	0.152 ± 0.0154	**0.0401**
Elongation	10.642 ± 3.2935	12.518 ± 3.4855	**0.0080**
Fragmentation index-6	5.4 × 10^− 4^ ± 3.6 × 10^− 4^	4.6 × 10^− 4^ ± 4.3 × 10^− 4^	0.10215
Fragmentation index-18	1.1 × 10^− 4^ ± 1.0 × 10^− 4^	8.0 × 10^− 5^ ± 1.4 × 10^− 4^	**0.00126**
Fragmentation index-26	1.1 × 10^− 4^ ± 1.0 × 10^− 4^	8.0 × 10^− 5^ ± 1.4 × 10^− 4^	**0.00137**

SA/V, surface area-to-volume; Std, standard deviation

Pearson correlation analysis between morphogeometric features and PLS-DA-derived LV1 was summarized in Table 4. Features with correlation values 0.4≤|r| <0.7 were classified as moderate correlations, while those with|r| ≥ 0.7 were classified as strong correlations. Among geometric features, volume, gradient mean of thickness, and gradient standard deviation of thickness demonstrated relatively strong correlations with LV1 (|r| ≥ 0.7). Among shape features, Fragmentation Index-18 showed the highest correlation with LV1 (*r* = 0.38 for controls, *r* = 0.52 for T2D participants). To enhance robustness, features exhibiting moderate to strong correlations (|r| ≥ 0.4) with LV1 were selected. A Random Forest classifier was employed to assess the ability of selected features to identify T2D-specific EAT characteristics, achieving an AUC of 0.703.

**Table 4:** Correlation of geometric and shape features with latent variable 1 (LV1) in T2D and control groups and selection of key features

Geometric features	Control	T2D	Selected
Volume (ml)	0.77	-0.85	√
SA/V (mm^− 1^)	-0.39	0.58	
Average thickness (mm)	0.45	-0.48	√
Max thickness (mm)	0.47	-0.44	√
Thickness variance	0.41	-0.38	
Gradient mean of thickness	0.77	-0.85	√
Gradient max of thickness	0.45	-0.45	√
Gradient std of thickness	0.71	-0.77	√

Shape features	Control	T2D	Selected
Compactness	-0.24	0.06	
Sphericity	-0.24	0.06	
Elongation	0.39	-0.23	
Fragmentation index-6	-0.37	0.56	
Fragmentation index-18	-0.38	0.52	√
Fragmentation index-26	-0.38	0.51	√

SA/V, surface area-to-volume; Std, standard deviation

## Discussion

This study developed a deep learning-based framework for automated EAT segmentation and morphogeometric profiling from Dixon MRI and applied it to characterize differences between individuals with T2D and controls. Incorporating multiple Dixon phase images improved segmentation accuracy compared with using only standard FP and WP images, highlighting the complementary imaging information provided by each phase. Morphogeometric analysis revealed that EAT in T2D participants was characterized by larger volume, lower SA/V ratio, greater thickness, and increased spatial heterogeneity. These features suggest structural remodeling of EAT in T2D [37–39]. Shape descriptors further demonstrated greater elongation and reduced fragmentation, suggesting more consolidated fat deposition in T2D. Importantly, moderate to strong correlations between key morphogeometric features and LV1 from statistical shape analysis support the biological relevance of these differences (Appendix Figure S2). A Random Forest classifier based on these features demonstrated good discrimination of T2D-associated EAT characteristics, suggesting potential utility of morphogeometric features as imaging biomarkers.

In our study, the observed geometric and shape differences in the T2D group provide morphological evidence supporting the established pathophysiological role of EAT in metabolic disease. These changes reflect structural remodeling processes that have been linked to proinflammatory and profibrotic activity within EAT in T2D [2]. T2D patient often show increased EAT volume and thickness, which have been associated with adverse cardiac remodeling and impaired cardiac function [2, 8]. Additionally, the presence of this metabolically active adipose stores that surround epicardial coronary arteries could contribute to the inflammatory burden [40–42]. In a study of over 400 diabetic patients, Cosson et al. found that EAT volume was independently associated with coronary artery calcification [43], and Christensen et al. summarized that studies have consistently reported both increased EAT in individuals with T2D and its association with CVD, suggesting that EAT may serve as a potential mechanistic link between diabetes and cardiovascular complications [44].

Our findings are consistent with and extend prior work by demonstrating spatially heterogeneous remodeling patterns of EAT in individuals with T2D. For example, Hu et al. reported that EAT thickness heterogeneity, quantified by the kurtosis of regional thickness across four axial heart slabs, was associated with major adverse cardiovascular events [45]. Song et al. also demonstrated that EAT thickness in the left atrioventricular groove was significantly higher in individuals with T2D than in non-diabetic controls [27]. Rather than focusing on specific anatomical regions, we employed whole-heart voxel-wise local thickness mapping to characterize global spatial non-uniformity of EAT. To quantify this heterogeneity, we introduced gradient-based thickness metrics—namely, the mean, maximum, and standard deviation of the local thickness gradient—as well as a fragmentation index to assess the degree of spatial disconnection. T2D participants exhibited 28.0%, 12.7%, and 17.7% higher values in gradient mean, maximum, and standard deviation, respectively, compared to controls, suggesting that fat accumulation in T2D is spatially non-uniform, with localized increases in certain regions potentially linked to enhanced metabolic or inflammatory activity. In addition, fragmentation indices (computed at − 18 and − 26 connectivity thresholds) were 27.3% lower in T2D, indicating that EAT in these individuals was less spatially fragmented. This likely reflects the combined effect of greater EAT volume and thickness in T2D, accompanied by expanded surface continuity and increased connectedness of fat depots across the myocardium.

Previous studies focusing on global EAT thickness distribution have primarily relied on manual measurements at selected anatomical landmarks [46]. For instance, Brigham et al. conducted an ex vivo analysis of 80 perfusion-fixed human hearts, quantifying regional EAT thickness at 51 predefined points via 3D reconstruction, but this analysis was limited to the ventricular surface [28]. Abbara et al. developed an EAT thickness mapping system including 32 segments to assist in transepicardial arrhythmia ablation [47]; however, its complexity and dependence on manual segmentation may hinder scalability and reproducibility. In contrast, our method enables comprehensive, fully automated, spatially resolved estimation of thickness at every voxel, facilitating population-level morphogeometric profiling without the need for manual input. This approach provides a scalable and reproducible foundation for integrating morphogeometric and spatial distribution metrics into cardiometabolic risk stratification models.

**Limitations**.

Several limitations should be acknowledged. First, this study employed input-level fusion to combine multi-phase Dixon images; more sophisticated fusion strategies, such as layer-level fusion, may better capture cross-modal interactions and further improve segmentation performance. Second, global EAT was analyzed without chamber-specific or region-specific mapping. Extending the framework to enable chamber-wise segmentation could reveal localized remodeling patterns and improve anatomical specificity. Moreover, incorporating regional EAT metrics—such as thickness or volume at specific anatomical landmarks—may further enhance clinical risk prediction. Third, the study was limited by the size and clinical depth of the dataset. The clinical relevance of the identified features may be constrained by the absence of clinical variables such as disease duration, treatment history, and cardiovascular outcomes. To strengthen clinical interpretation and external validity, future studies should incorporate larger, more diverse, and clinically well-annotated cohorts, including data from multiple centers. However, multicenter data aggregation will inevitably introduce additional challenges, such as variations in imaging protocols and population, leading to potential domain shifts. To address this, we will explore strategies such as domain adaptation and transfer learning to improve the generalizability and robustness of the proposed framework across heterogeneous imaging environments.

## Conclusions

In conclusion, this study presented a framework for comprehensive EAT analysis from MRI and systematically investigated the spatial distribution and morphogeometric differences of EAT between individuals with T2D and controls. The results provide evidence of distinct EAT remodeling in T2D, highlighting the potential of shape- and geometry-based features as imaging biomarkers of cardiometabolic risk. These findings support further studies to explore the broader clinical significance of EAT in risk stratification and disease monitoring.

## Electronic supplementary material

Below is the link to the electronic supplementary material.

Supplementary Material 1

## Data Availability

The datasets are not publicly available because they are part of a private dataset not yet approved for public release. However, the source code used for model training and analysis will be made publicly available upon article acceptance.

## Abbreviations

AI: Artificial intelligence
ASSD: Average symmetric surface distance
AUC: Area under the curve
CT: Computed tomography
CNN: Convolutional neural network
CVD: Cardiovascular disease
DSC: Dice similarity coefficient
EAT: Epicardial adipose tissue
FP: Fat phase
HD95: 95% Hausdorff distance
IP: In-phase
LV1: Latent variable 1
LV2: Latent variable 2
MRI: Magnetic resonance imaging
OP: Out-of-phase
PLS-DA: Partial least squares discriminant analysis
ROC: Receiver operating characteristic
SA/V: Surface area-to-volume
SDM: Signed distance map
T2D: Type 2 diabetes
VIP: Variable importance in projection. WP = water phase
3D: Three-dimensional
