# Supplementary material for: AI-Driven segmentation and morphogeometric profiling of epicardial adipose tissue in type 2 diabetes
Source: Cardiovasc Diabetol. 2025 Jul 18;24:294. doi: 10.1186/s12933-025-02829-y (PMC12275356; doi:10.1186/s12933-025-02829-y)
Supplement: Supplementary file 1 — Supplementary Material 1 [file 12933_2025_2829_MOESM1_ESM.docx]

**Supplemental Material**

**Appendix S1**

**Labeling Procedures**

Epicardial adipose tissue (EAT) ground truth segmentations were generated from cardiac 3D Dixon MRI datasets. The transversal image stacks were analysed using the open-source software MITK (version 2018.04.2) and Avizo software (version 2020.1). Manual segmentation was performed at end-diastole, starting from the most caudal slice where myocardium was visible and proceeding cranially to the first slice where the right branch of the pulmonary artery showed continuity [1]. The pericardium was first delineated on each slice to define the region of interest (ROI), ensuring exclusion of paracardial fat external to the parietal pericardium. Subsequently, within the ROI, the fat phase (FP) images were used as the primary reference for EAT segmentation. Image intensity thresholds were manually adjusted to suppress obvious noise and enhance adipose tissue visualization. EAT was then segmented in a pixel-by-pixel manner across slices. To minimize partial volume effects and misclassification, corresponding water phase (WP) images were cross-referenced for verification. Annotations were independently performed by two experts, each with more than three years of experience in cardiac MRI interpretation and atrial anatomy. To ensure labeling consistency and accuracy, a third expert reviewed all annotations. Any discrepancies were resolved through consensus discussions among the three experts.

**Appendix S2**

Fat map (FM) is included as an input channel to enhance the fat signal representation and is computed from the OP, FP, and WP phases using:

| FM =$f_{\mathrm{threshold}}(OP-WP+ FP)$ | (1) |
| --- | --- |

Here, the threshold function $f_{\mathrm{threshold}}$ is determined by combining Otsu's method with the mean grayscale value:

| Threshold =$(Otsu's value + mean value)/2$ | (2) |
| --- | --- |

Pixels with intensities lower than the threshold are considered as background. FM has been demonstrated in previous study to enhance EAT segmentation performance by delineating fat-specific structures more effectively [2].

**Appendix S3**

To quantify the distance between the predicted and ground truth distance maps, both L1 and L2 norm-based loss functions were considered.

For the L1 norm, the distance loss is defined as:

| $\mathcal{L}_{\mathrm{dist}}^{L1}=\frac{\left\Vert P_{dis}-G_{dis} \right\Vert_{1}}{numel(P_{dis})}$ | (3) |
| --- | --- |

where $P_{dis}$ and $G_{dis}$ represent the predicted and ground truth signed distance maps (SDMs), respectively, and $numel(P_{dis})$ denotes the total number of elements $P_{dis}$. L1 loss encourages sparsity, which helps suppress minor noise and highlights critical structures in the distance map.

For the L2 norm, the distance loss is defined as:

| $\mathcal{L}_{\mathrm{dist}}^{L2}=\frac{\left\Vert P_{dis}-G_{dis} \right\Vert_{2}^{2}}{numel(P_{dis})}$ | (4) |
| --- | --- |

The L2 norm penalizes larger prediction errors more heavily due to the quadratic operation, thereby prioritizing the correction of large deviations during optimization. Its continuous and smooth gradient promotes more stable and efficient convergence during training.

**Appendix S4**

**Data preprocessing and point-cloud shape alignment**

Point cloud data are extracted from epicardial adipose tissue (EAT) volumes and undergo a rigorous preprocessing pipeline to ensure spatial consistency and comparability across samples. Point clouds are used due to their flexibility in capturing complex geometric details, computational efficiency, and compatibility with statistical shape analysis. The preprocessing includes the following key steps:

***Alignment***: Each point cloud is first scaled and centered by positioning the centroid of its bounding box. The Iterative Closest Point (ICP) registration is applied to align the global spatial structures of all point cloud, eliminating positional and rotational differences across samples while preserving intrinsic geometric features.

***Non-Rigid Registration***: The Coherent Point Drift (CPD) algorithm is utilized for non-rigid point cloud registration [3], which models the source point set as a Gaussian mixture model and aligns it to the target set by maximizing the likelihood under a probabilistic framework. This approach enables smooth and coherent local deformations while preserving global geometric structure. Because CPD does not rely on mesh topology or structural continuity, it is well-suited for anatomical structures such as EAT, which may exhibit fragmented or irregular morphologies. The adaptive voxel downsampling ensures efficient and accurate alignment without compromising the quality of shape analysis.

***Fitting***: The deformed point cloud is mapped to a unified bounding box for feature correspondence. A KD-tree search assigns each point to its nearest template point, identifying EAT points while treating unmatched regions as background. The final points are indexed by the bounding box for consistent statistical analysis.

**Appendix S5**

Fragmentation indexes based on 6-, 18-, and 26-connectivity were computed to quantify EAT uniformity or fragmentation based on the ratio of EAT connectivity to the total volume:

| $\mathrm{Fragmentation} \mathrm{Index}=\frac{Number of Connected Components}{EAT Volume}$ | (5) |
| --- | --- |

These indexes differ by connectivity definitions: index-6 (face adjacency), index-18 (face and edge adjacency), and index-26 (face, edge, and corner adjacency).

**Appendix S6**

Model training and evaluation were performed on an NVIDIA Tesla V100 GPU. Input images were centrally cropped to 320 × 320 pixels, and a batch size of 4 was used. Data augmentation techniques, including random rotation, elastic deformation, and intensity shifts, were applied to enhance the robustness and generalization capability of the model. The network was trained using the AdamW optimizer with an initial learning rate of 0.0001 for 150 epochs. For comparative analysis, EAT-Seg was benchmarked against two baseline models, ResUNet [4] and UNet++ [5]. To ensure fair comparison, all models were trained and evaluated using the same dataset partitions, data augmentation strategies, and evaluation metrics. Model performance was assessed across all combinations of different Dixon MRI phase images.

**Table S1.** Clinical characteristics and comparison between control and T2D groups

|  | **Control (N=45)** | **T2D (N=45)** | **P-Value** |
| --- | --- | --- | --- |
| Age (years) | 60 (50 - 64) | 60 (50 - 64) | 0.76 |
| Sex (female) | 16 (35.6%) | 16 (35.6%) | - |
| Self-reported cardiac disease | 3 (6.7%) | 8 (17.8%) | - |
| BMI | 25.8 (19.5 - 33.1) | 29.95 (20.3 - 36.0) | < 0.001 |
| Heart rate (BPM) | 66 (43 - 89) | 69 (51 - 99) | < 0.05 |
| LVEF (%) | 60 (38 - 72) | 60 (26 - 79) | 0.28 |
| LV mass index (g/m2) | 59.4 (43.9 - 79.0) | 61.2 (41.9 - 108.2) | 0.29 |

Data shown as median (minimum - maximum) or n (%). T2D, type 2 diabetes; LVEF (%), left ventricular ejection fraction as a percentage; BMI, body mass index; BPM, beats per minute; LV, left ventricle.

**Table S2.** Summary of published studies on deep learning-based EAT/PAT segmentation

| **Study** | **Data Modality** | **Data Source** | **Data Quantity** | **Resolution** | **Intra-spacing(mm^2^)** | **Inter-spacing(mm)** |
| --- | --- | --- | --- | --- | --- | --- |
| [6] | Non-contrast CT | Private | 250 | 512 × 512 | 0.684 × 0.684 | 2.5 or 3 |
| [7] | Non-contrast CT | Private | 850 | 512 × 512 | (0.3007 to 0.6836)^2^ | 1.5 to 3 |
| [8] | Non-contrast CT | Private | 2068 | - | - | - |
| [9] | Non-contrast CT | Private | 103 | 512 × 512 | (0.683 to 1.367)^2^ | 1.25 or 3.75 |
| [10] | Non-contrast CT | Private | 70 | 512 × 512 | - | 2.5 |
| [11] | Non-contrast CT | Public | 20 | 512 × 512 | - | - |
| [12] | Non-contrast CT | Private | 1811 | 512 × 512 | - | 3 |
| [13] | Non-contrast CT | Private | 93 | 512 × 512 | (0.66 to 0.86)^2^ | 2.5 |
| [14] | Non-contrast CT | Private | 8781 | - | - | - |
| [15] | Contrast-enhanced CT | Private | 108 | 512 × 512 | - | 3 |
| [16] | Contrast-enhanced CT | Private | 3720 | - | - | - |
| [17] | Contrast-enhanced CT | Private | 200 | 512 × 512 | 0.28 × 0.28 | 0.75 |
| [18] | Contrast-enhanced CT | Private | 40 | 512 × 512 | 0.28 × 0.28 | 0.5 |
| [19] | Non-contrast CT | Public | 220 | 256 × 256 | 0.98 × 0.98 | 2.5 |
|  | Contrast-enhanced CT | Public | 40 | 256 × 256 | (0.61 to 0.98)^2^ | 0.625 |
| [20] | MRI | Public | 150 | (240 to 352)^2^ | (0.868 to 1.25)^2^ | 7 |
| [21] | MRI | Private | 100 | - | (1.3 to 1.8)^2^ | 6 |
| [22] | MRI | Private | 730 | (148 to 224)^2^ | - | 8 |
| [23] | MRI | Public | 150 | - | (1.37 to 1.68)^2^ | 5 to 10 |
|  | MRI | Private | 71 | 256 × 166 | - | 8 |

‘–’ indicates that no relevant information is given; EAT, epicardial adipose tissue; PAT, pericardial adipose tissue.


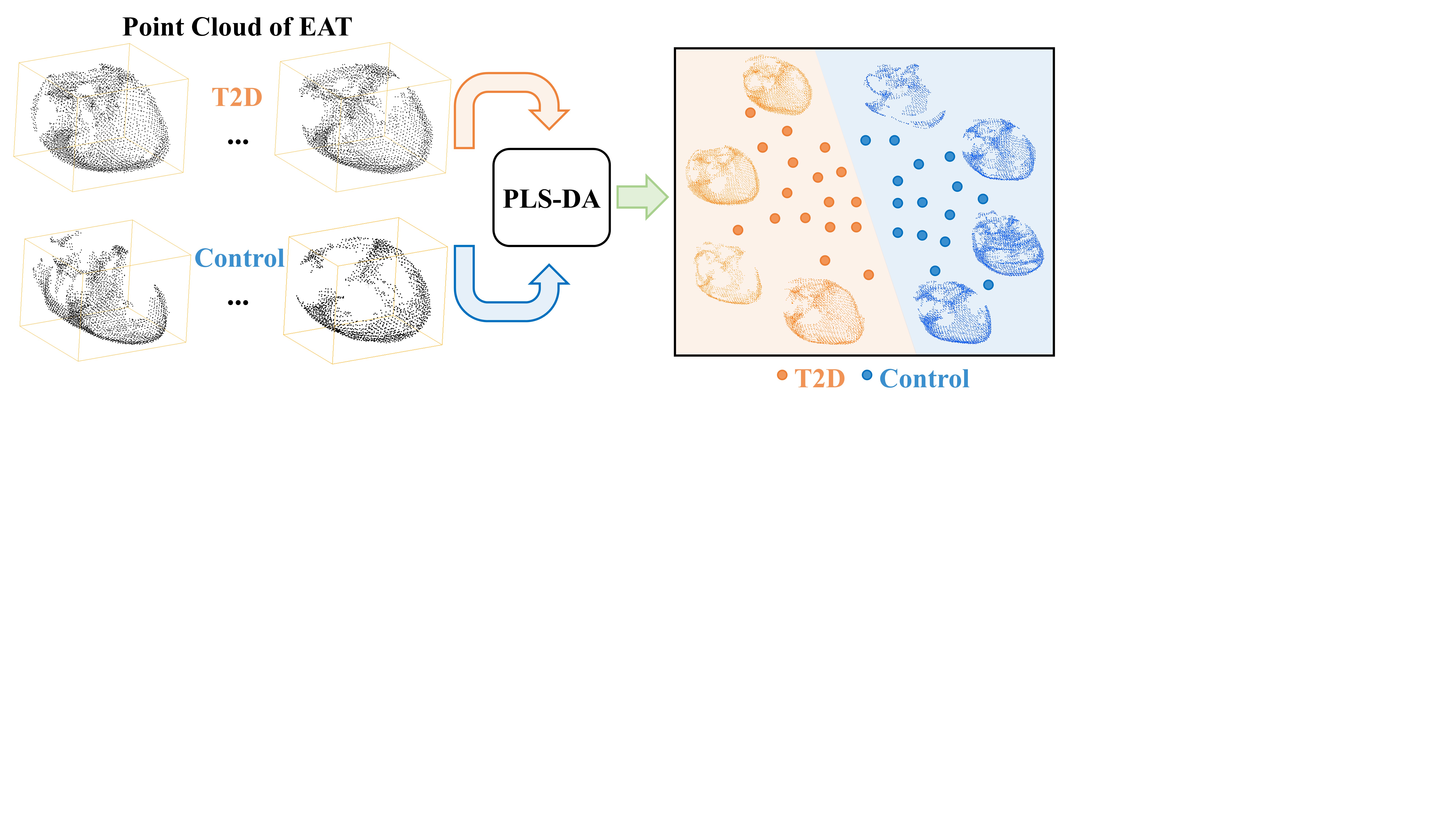


**Figure S1.** Partial least squares discriminant analysis (PLS-DA)-based shape analysis of epicardial adipose tissue (EAT) in type 2 diabetes (T2D) and control groups. Point cloud representations of EAT are derived from segmented EAT volumes following a series of preprocessing steps, including normalization, rigid registration (ICP), adaptive voxel downsampling, and non-rigid registration (CPD). The processed point clouds are then analyzed using PLS-DA, a multivariate statistical method that projects high-dimensional shape data onto a lower-dimensional latent space while optimizing group separation.


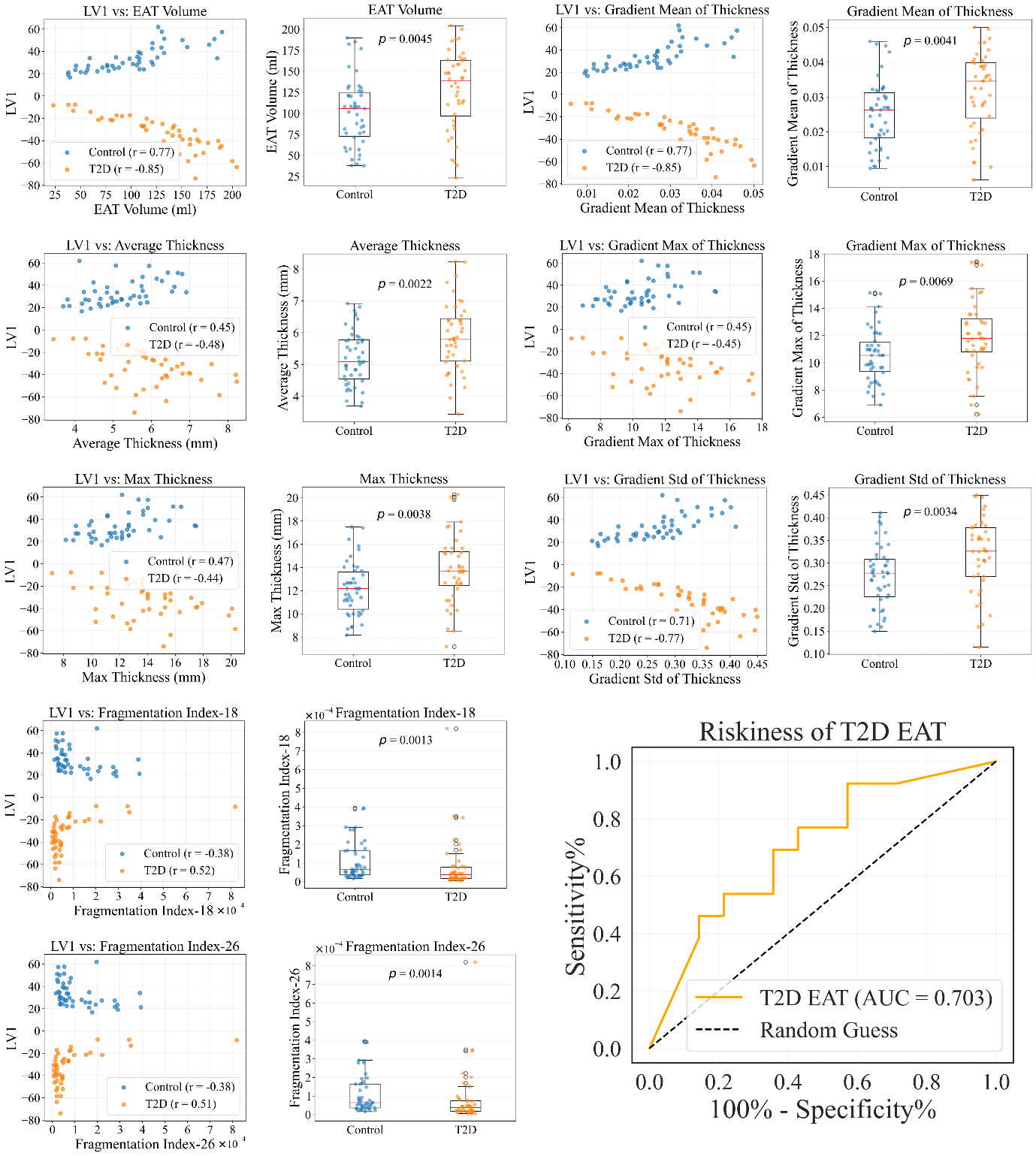


**Figure S2.** Correlation between morphogeometric features and latent variable 1 (LV1) in type 2 diabetes (T2D) and control groups. Scatter plots illustrate the relationships between selected geometric and shape features with LV1, with higher correlations observed for epicardial adipose tissue (EAT) volume, thickness metrics, and gradient features. The receiver operating characteristic (ROC) curve (bottom right) demonstrates the classification performance of the Random Forest model, achieving an area under the curve (AUC) of 0.703 in distinguishing T2D and control groups based on the selected features.

**References**

1. Edin C, Ekstedt M, Scheffel T, Karlsson M, Swahn E, Östgren CJ, et al. Ectopic fat is associated with cardiac remodeling—A comprehensive assessment of regional fat depots in type 2 diabetes using multi-parametric MRI. Front Cardiovasc Med. 2022;9:813427.

2. Feng F, Carlhäll C-J, Tan Y, Agrawal S, Lundberg P, Bai J, et al. FM-Net: A Fully Automatic Deep Learning Pipeline for Epicardial Adipose Tissue Segmentation. In: Camara O, Puyol-Antón E, Sermesant M, Suinesiaputra A, Tao Q, Wang C, et al., editors. Statistical Atlases and Computational Models of the Heart Regular and CMRxRecon Challenge Papers [Internet]. Cham: Springer Nature Switzerland; 2024 [cited 2025 Apr 3]. p. 88–97. Available from: https://link.springer.com/10.1007/978-3-031-52448-6_9

3. Myronenko A, Xubo Song. Point Set Registration: Coherent Point Drift. IEEE Trans Pattern Anal Mach Intell. 2010;32:2262–75.

4. Kerfoot E, Clough J, Oksuz I, Lee J, King AP, Schnabel JA. Left-Ventricle Quantification Using Residual U-Net. In: Pop M, Sermesant M, Zhao J, Li S, McLeod K, Young A, et al., editors. Statistical Atlases and Computational Models of the Heart Atrial Segmentation and LV Quantification Challenges [Internet]. Cham: Springer International Publishing; 2019 [cited 2025 Feb 19]. p. 371–80. Available from: https://link.springer.com/10.1007/978-3-030-12029-0_40

5. Zhou Z, Rahman Siddiquee MM, Tajbakhsh N, Liang J. UNet++: A Nested U-Net Architecture for Medical Image Segmentation. In: Stoyanov D, Taylor Z, Carneiro G, Syeda-Mahmood T, Martel A, Maier-Hein L, et al., editors. Deep Learning in Medical Image Analysis and Multimodal Learning for Clinical Decision Support [Internet]. Cham: Springer International Publishing; 2018 [cited 2025 Feb 19]. p. 3–11. Available from: https://link.springer.com/10.1007/978-3-030-00889-5_1

6. Commandeur F, Goeller M, Betancur J, Cadet S, Doris M, Chen X, et al. Deep Learning for Quantification of Epicardial and Thoracic Adipose Tissue From Non-Contrast CT. IEEE Trans Med Imaging. 2018;37:1835–46.

7. Commandeur F, Goeller M, Razipour A, Cadet S, Hell MM, Kwiecinski J, et al. Fully Automated CT Quantification of Epicardial Adipose Tissue by Deep Learning: A Multicenter Study. Radiology: Artificial Intelligence. 2019;1:e190045.

8. Eisenberg E, McElhinney PA, Commandeur F, Chen X, Cadet S, Goeller M, et al. Deep Learning–Based Quantification of Epicardial Adipose Tissue Volume and Attenuation Predicts Major Adverse Cardiovascular Events in Asymptomatic Subjects. Circ: Cardiovascular Imaging. 2020;13:e009829.

9. Qu J, Chang Y, Sun L, Li Y, Si Q, Yang M-F, et al. Deep Learning-Based Approach for the Automatic Quantification of Epicardial Adipose Tissue from Non-Contrast CT. Cogn Comput. 2022;14:1392–404.

10. Hoori A, Hu T, Al-Kindi S, Rajagopalan S, Wilson DL. Automatic Deep Learning Segmentation and Quantification of Epicardial Adipose Tissue in Non-Contrast Cardiac CT scans. 2021 43rd Annual International Conference of the IEEE Engineering in Medicine & Biology Society (EMBC) [Internet]. Mexico: IEEE; 2021 [cited 2024 Oct 31]. p. 3938–42. Available from: https://ieeexplore.ieee.org/document/9630953/

11. Zhang Q, Zhou J, Zhang B, Jia W, Wu E. Automatic Epicardial Fat Segmentation and Quantification of CT Scans Using Dual U-Nets With a Morphological Processing Layer. IEEE Access. 2020;8:128032–41.

12. Molnar D. Artificial intelligence based automatic quantification of epicardial adipose tissue suitable for large scale population studies. Scientific Reports. 2021;

13. Hoori A, Hu T, Lee J, Al-Kindi S, Rajagopalan S, Wilson DL. Deep learning segmentation and quantification method for assessing epicardial adipose tissue in CT calcium score scans. Sci Rep. 2022;12:2276.

14. Miller RJH, Shanbhag A, Killekar A, Lemley M, Bednarski B, Van Kriekinge SD, et al. AI-derived epicardial fat measurements improve cardiovascular risk prediction from myocardial perfusion imaging. npj Digit Med. 2024;7:24.

15. Tang K-X, Liao X-B, Yuan L-Q, He S-Q, Wang M, Mei X-L, et al. An enhanced deep learning method for the quantification of epicardial adipose tissue. Sci Rep. 2024;14:24947.

16. West HW, Siddique M, Williams MC, Volpe L, Desai R, Lyasheva M, et al. Deep-Learning for Epicardial Adipose Tissue Assessment With Computed Tomography. JACC: Cardiovascular Imaging. 2023;16:800–16.

17. He X, Guo BJ, Lei Y, Wang T, Fu Y, Curran WJ, et al. Automatic segmentation and quantification of epicardial adipose tissue from coronary computed tomography angiography. Phys Med Biol. 2020;65:095012.

18. He X, Guo B, Lei Y, Wang T, Liu T, Curran WJ, et al. Automatic epicardial fat segmentation in cardiac CT imaging using 3D deep attention U-Net. In: Landman BA, Išgum I, editors. Medical Imaging 2020: Image Processing [Internet]. Houston, United States: SPIE; 2020 [cited 2023 Oct 9]. p. 84. Available from: https://www.spiedigitallibrary.org/conference-proceedings-of-spie/11313/2550383/Automatic-epicardial-fat-segmentation-in-cardiac-CT-imaging-using-3D/10.1117/12.2550383.full

19. Siriapisith T, Kusakunniran W, Haddawy P. A 3D deep learning approach to epicardial fat segmentation in non-contrast and post-contrast cardiac CT images. PeerJ Computer Science. 2021;7:e806.

20. Chen S, An D, Feng C, Bian Z, Wu L-M. Segmentation of Pericardial Adipose Tissue in CMR Images: A Benchmark Dataset MRPEAT and a Triple-Stage Network 3SUnet. IEEE Trans Med Imaging. 2023;42:2386–99.

21. Daudé P, Ancel P, Confort Gouny S, Jacquier A, Kober F, Dutour A, et al. Deep-Learning Segmentation of Epicardial Adipose Tissue Using Four-Chamber Cardiac Magnetic Resonance Imaging. Diagnostics. 2022;12:126.

22. Guglielmo M, Penso M, Carerj ML, Giacari CM, Volpe A, Fusini L, et al. DEep LearnIng-based QuaNtification of epicardial adipose tissue predicts MACE in patients undergoing stress CMR. Atherosclerosis. 2024;397:117549.

23. Zhao K, Liu Z, Liu J, Zhou J, Liao B, Tang H, et al. Uncertainty-driven and Adversarial Calibration Learning for Epicardial Adipose Tissue Segmentation [Internet]. arXiv; 2024 [cited 2024 Oct 31]. Available from: http://arxiv.org/abs/2402.14349
